# Renal Stem Cells, Tissue Regeneration, and Stem Cell Therapies for Renal Diseases

**DOI:** 10.1155/2015/302792

**Published:** 2015-05-28

**Authors:** Benedetta Bussolati, Akito Maeshima, Janos Peti-Peterdi, Takashi Yokoo, Laura Lasagni

**Affiliations:** ^1^Center for Molecular Biotechnology, Department of Molecular Biotechnology and Health Sciences, 10126 Torino, Italy; ^2^Department of Medicine and Clinical Science, Gunma University Graduate School of Medicine, Maebashi, Gunma 371-8511, Japan; ^3^Departments of Physiology and Biophysics and Medicine, University of Southern California, Los Angeles, CA 90033, USA; ^4^Department of Internal Medicine, Jikei University School of Medicine, Tokyo 3-19-18, Japan; ^5^DENOTHE Center, Department of Clinical and Experimental Medicine, University of Florence, 50139 Florence, Italy

Kidney diseases are a global public health problem, with an incidence that has reached epidemic proportions and continues to climb in the USA and worldwide. This trend is projected to grow in correlation with the global rise in the aged population and the increasing prevalence of conditions that cause renal complications, such as cardiovascular disease, hypertension, and diabetes. Current treatment options for acute and chronic kidney disease include dialysis, which is also associated with substantial morbidity and mortality, and kidney transplantation, which is limited by the supply of compatible organs. Consequently, new methods to alleviate, cure, or prevent renal disease are urgently required to reduce the exponentially growing burden due to acute and chronic kidney disorders and offer alternative therapeutic options to improve patients' survival and quality of life. Several potential regenerative cell-based therapies for the treatment of renal failure are currently under development. The first one is the direct application of stem cells (SCs) to the diseased kidney, which relies on the inherent capabilities of SCs to differentiate, organize, and integrate into the existing tissues to restore function. To this aim different stem cell types have been investigated for their potential to contribute to renal regeneration. In their article “WNT/*β*-Catenin Signaling Is Required for Integration of CD_24+_ Renal Progenitor Cells into Glycerol-Damaged Adult Renal Tubules,” Z. Zhang et al. described how the endogenous canonical *β*-catenin/TCF pathway is reactivated during recovery from AKI and is required for the engraftment of exogenous CD24^+^ embryonic renal progenitor cells into damaged tubular areas upon injury. These events appear to recapitulate the WNT-dependent inductive process which drives primary nephrogenesis. Review article by F. O. Arcolino et al. entitled “Human Urine as a Noninvasive Source of Kidney Cells” summarizes the recent data regarding human urine-derived cells. Urine contains various types of kidney cells such as podocytes, proximal tubule cells, or renal stem/progenitor cells, which can be easily as well as noninvasively isolated and cultured* in vitro*. Although the characteristics of each cell population are not totally understood, this paper suggests the potential of urine-derived kidney cells as a tool of clinical application for the treatment of various kidney diseases. Stem cell therapy contributes for kidney regeneration not only through direct differentiation and replacement of injured cells but also by secretion of renoprotective or trophic factors. These data are summarized by K. Tsuji and S. Kitamura in their review article “Trophic Factors from Tissue Stem Cells for Renal Regeneration.”

Another strategy is based on the prospective design of a therapeutic approach focused on modulation of endogenous kidney regenerative properties by conventional chemical and biological agents able to modulate the activity of resident progenitor cells. Although understanding of regeneration of the nephron may have significance to explore new therapeutic approaches, the process of regeneration of nephron has yet been poorly understood. The review article “Atlas of Cellular Dynamics during Zebrafish Adult Kidney Regeneration” by K. K. McCampbell et al. provides an essential foundation for future work aimed at elucidating the mechanisms that regulate kidney regeneration following acute kidney injury using adult zebrafish, which maintains the regenerative ability. Akito Maeshima et al. in their review “Diverse Cell Populations Involved in Regeneration of Renal Tubular Epithelium following Acute Kidney Injury” describe recent advances in understanding the regeneration mechanisms of renal tubules, particularly the characteristics of various cell populations contributing to tubular regeneration and highlight the targets for the development of regenerative medicine for treating kidney diseases in humans. Additional information on the renal regeneration capacity could be inferred from studies on kidney organ culture. The review article “Organ* In Vitro* Culture: What Have We Learned about Early Kidney Development?” by A. Rak-Raszewska et al. provides an introduction to the organ culture method and a summary of the progress in the field of kidney developmental biology based on it. Finally, a great advance on the comprehension of the mechanisms of renal regeneration, in particular podocyte regeneration, and on the role of putative population of renal stem/progenitor cells could be obtained from transgenic mouse models, as summarized by D. Lombardi and Laura Lasagni in their review “Transgenic Strategies to Study Podocyte Loss and Regeneration.” Finally, a number of different approaches have been applied toward tissue engineering of the kidney as a mean to replace renal function. S. Yamanaka and Takashi Yokoo in their review article “Current Bioengineering Methods for Whole Kidney Regeneration” summarized recent researches involving the use of renal stem cells and renal bioengineering to regenerate functional whole kidneys* de novo*.

The editors hope that the original and review articles integrated in this special issue provide more insights into the advancements and challenges faced by this rapidly expanding field of regenerative medicine and will be helpful and educational for interested readers.

*Benedetta Bussolati* *Benedetta Bussolati* *Akito Maeshima* *Akito Maeshima* *Janos Peti-Peterdi* *Janos Peti-Peterdi* *Takashi Yokoo* *Takashi Yokoo* *Laura Lasagni* *Laura Lasagni*

